# Self-induced mechanical stress can trigger biofilm formation in uropathogenic *Escherichia coli*

**DOI:** 10.1038/s41467-018-06552-z

**Published:** 2018-10-05

**Authors:** Eric K. Chu, Onur Kilic, Hojung Cho, Alex Groisman, Andre Levchenko

**Affiliations:** 1 Department of Biomedical Engineering, Yale University, New Haven, CT 06520 USA; 2 Yale Systems Biology Institute, Yale University, West Haven, CT 06516 USA; 3 Department of Biomedical Engineering, The Johns Hopkins University School of Medicine, Baltimore, MD 21205 USA; 4 Department of Physics, University of California, San Diego, La Jolla, CA 92093 USA

## Abstract

Bacterial biofilms represent an important medical problem; however, the mechanisms of the onset of biofilm formation are poorly understood. Here, using new controlled methods allowing high-throughput and reproducible biofilm growth, we show that biofilm formation is linked to self-imposed mechanical stress. In growing uropathogenic *Escherichia coli* colonies, we report that mechanical stress can initially emerge from the physical stress accompanying colony confinement within micro-cavities or hydrogel environments reminiscent of the cytosol of host cells. Biofilm formation can then be enhanced by a nutrient access-modulated feedback loop, in which biofilm matrix deposition can be particularly high in areas of increased mechanical and biological stress, with the deposited matrix further enhancing the stress levels. This feedback regulation can lead to adaptive and diverse biofilm formation guided by the environmental stresses. Our results suggest previously unappreciated mechanisms of the onset and progression of biofilm growth.

## Abstract (web summary)

Bacterial biofilms are an increasingly important medical problem but the mechanisms by which they develop remain largely unknown. Here, using a high-throughput approach, the authors show that biofilm formation is linked to self-imposed mechanical stress.

## Introduction

Under stressful conditions, bacterial cells can actively seek out protective, spatially isolated niches, such as micro-cavities in the complex mechanical micro-environments^1,2^ or the cytosolic compartments of host cells^3,4^. There, they can be shielded from adverse effects of the environment and grow to high densities. The resultant tight cell packing leads to various forms of collective behavior, shaped by both biological responses and mechanical effects^5–10^. An example of a tightly packed, spatially confined population is the intracellular bacterial colonies (IBCs) of *E. coli* growing within infected epithelial cells. The recurrence and persistence of bacterial urinary tract infections (UTIs) is ascribed to the ability of *E. coli* cells to invade epithelial cells lining the lumens of the urinary tract, to form tightly packed IBCs, and to disperse out of the host^3,4^. These IBCs are structured as biofilms that have pod-like three-dimensional (3-D) appearances and express various extracellular matrix components, including elements of exopolysaccharide (EPS) and protein fibers (e.g., curli)^11,12^. This property affords the bacterial cells dual protection from the host defenses: shielding of the potential immune triggers within the host cell cytosol and suppression of the effect of antibiotic treatment due to more effective penetration barriers^13,14^. On the other hand, tight packing in the host cells and the expression of the matrix components can enhance stresses on the colony due to mechanical constraining of colony growth and declining access to nutrients. Mechanical interactions are known to be crucial for the transition from 2 to 3-D bacterial colonies^15,16^, but it is unclear how a tightly packed, confined population would respond to an increasing buildup of self-imposed mechanical stress. The interplay between the benefits and disadvantages of the intracellular biofilm formation has likely defined the evolutionary pressure on both bacterial invasion and the host responses. However, the complexity of the interaction between the host and IBCs has restricted our understanding of the onset of the formation of biofilm pod-like structures and the associated costs and benefits.

Here, we modeled the emergence of IBCs using a series of simple but highly controlled environmental culture conditions, focusing in particular on the role of mechanical stresses stemming from colony expansion within confining environments. Strikingly, we observed that self-imposed mechanical stress can trigger the transition to a biofilm-like phenotype with antibiotic tolerance in uropathogenic *E. coli*. Further analysis using a combination of mathematical modeling and experimental observations suggested a number of feedback mechanisms controlling the interplay between mechanical stress and biofilm formation, and cell survival under stressful conditions. These results shed light on the mechanisms and consequences of biofilm formation and antibiotic tolerance during uropathogenic infections and, potentially, other instances of bacterial biofilms.

## Results

### Confined growth leads to self-generated pressure buildup and stress response

**Fig. 1:**
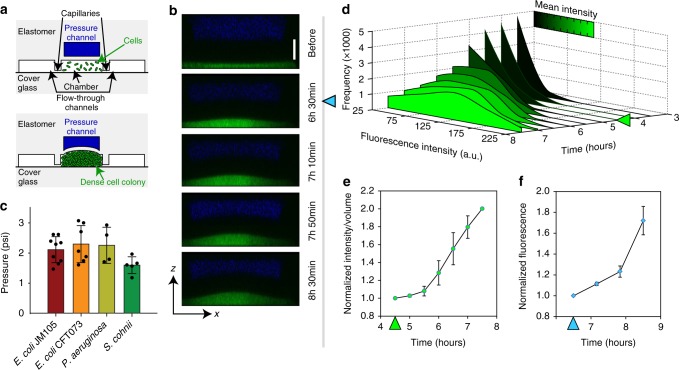
Pressure buildup as a result of confined growth leads to biochemical stress response. **a** Schematic cross-section of the microfluidic device, illustrating the deformation of the flexible roof due to bacterial colony growth. **b** The deformation of the roof [black area between blue (Alexa Fluor 647) and green (GFP-expressing *E. coli*) layers] is visualized with CLSM. Scale bar for the vertical dimension, 20 μm. **c** Maximum pressures produced by different bacteria; *E. coli* JM105 (*n* = 9); *E. coli* CFT073 (*n* = 7); *P. aeruguinosa* (*n* = 4); *S. cohnii* (*n* = 5). **d** Distributions of *rpoH* expression-reporting GFP fluorescence intensity per pixel in a WFM image of a chamber (in arbitrary units ranged 25 to 225) at various time points, starting 3.5 h after seeding cells into the chamber. The arrow (*t* = 4.5 h) indicates when the chamber is completely filled with cells. The color of the plot shows the mean intensity at the corresponding time point. **e** The integral fluorescence of bacterial colony in the chamber divided by the chamber volume measured at the same time point. The data is normalized to the time point just before the roof deformation becomes detectable (*t* = 4.5 h in Fig. 1d) (*n* = 3). **f** GFP expression due to *rpoH* upregulation measured from *xy*-sections of confocal imaging. The data was normalized to the time point when roof deformation started to become measurable (*t* = 6.5 h in Fig. 1b, indicated by the blue arrow) (*n* = 3). Error bars are SD

Previously, we have shown that bacteria can be cultured in microfluidic chambers with boundaries permeable to nutrients and metabolites, but largely impassable to cells, leading to dense cell packing^17^. This type of culture provides spatial confinement and can serve as a model for understanding the effects of self-imposed mechanical stress on the expanding bacterial colonies. However, the rigid boundaries of the culture chambers prevent the precise evaluation of the pressure generated by the cells as well as the effects of continued growth with mechanical stresses in the colony. To assess the mechanical stresses in bacterial colonies, we modified the design of the device by making the roofs of the culture chambers into elastic boundaries. The modified microfluidic device (Fig. 1a, Supplementary Fig. 1a and Supplementary Movie 1) had an additional layer of channels on top of the culture chambers, separated from the chambers by a thin layer of polydimethylsiloxane (PDMS) which functioned as a deformable roof. Because of the high flexibility of PDMS, the roofs were expected to deform under forces exerted by bacterial colonies growing in the chambers. We applied a range of pressures to the growth chamber layer that was filled with a green fluorescent dye (while maintaining the top channels filled with a red fluorescent dye at the atmospheric pressure) and visualized the shapes of the roofs via 3-D reconstruction of the fluorescence distribution captured using confocal laser scanning microscopy (CLSM) (Supplementary Fig. 1b). By measuring the upward displacements of the roofs near their centers (where the displacements were maximal), we calibrated the roof deformations as a function of applied pressures in chambers with different sizes, and used this calibration to evaluate the pressures generated by colonies growing in these chambers. The data points on the maximal roof displacement vs. pressure calibration plots (Supplementary Fig. 1c) were in agreement with linear dependences predicted by mathematical modeling (Methods and Supplementary Fig. 2), which was based on a plate-theory model^18^.

In our experiments, bacterial colonies completely filled the chambers five to seven hours after cell loading. At this point, deformations of the chamber roofs became detectable (Fig. 1b and Supplementary Movie 2), and we began measuring the pressure exerted by the growing colonies upon the roofs (as determined using the calibration of the roof displacement vs. pressure; Supplementary Fig. 3). We measured pressure values for multiple bacterial species, including uropathogenic strain of *E. coli* (CFT073), cystic fibrosis-associated *P. aeruginosa* and a gram-positive spherical-shape *S. cohnii*. The shapes of the deformed roofs were largely the same for all chambers of a given size and for all strains, in agreement with the plate-theory model (Supplementary Fig. 4 and Supplementary Movie 3). All strains were capable of generating pressures greater than ~1.5 psi (~10 kPa; Fig. 1c). For an estimated cell footprint of ~1 µm^2^, this pressure corresponds to a force of ~1 nN. The pressure of 1.5 psi is comparable to the levels required to induce osmotic bursting of eukaryotic cells (e.g., ~1.0 psi, ~6.9 kPa for alveolar epithelial cells and ~0.6 psi, ~4.1 kPa for red blood cells)^19,20^. These results suggest that expanding bacterial colonies are capable of generating forces sufficient to rupture an intact membrane of the host-cell from inside, adding the mechanical bursting out of a host cell to the list of previously proposed possible dispersal mechanisms^21–23^. On the other hand, the pressures at the chamber roofs were lower than the estimated turgor pressure within bacterial cells (4.4–14.5 psi, 30–100 kPa)^24^, which is often considered as the potential maximum pressure needed to restrict bacterial growth. The colony growth arrest at such relatively low pressures at the boundary (roof) suggests that the arrest may not be solely due to the inability of individual cells at the roof to push against the roof any stronger, and that the mechanism of the arrest may involve some adaptive response by the colonies to an increasing environmental stress. In this regard, it is worth noting that the dynamics of IBCs are somewhat similar, with the initial rapid expansion followed by a growth arrest^25^, and that the pressure at the boundaries of the growth-arrested IBCs must be well below the bacterial turgor pressures (because otherwise the host cell would burst). One possible reason for additional resistance to colony expansion is the increased viscosity and solidification of the local cell environment due to potential formation of biofilm-like features. We thus set out to further characterize the response of the colony itself to the increasing mechanical stress generated by growth against a deforming elastic boundary.

A common cellular response to stresses of various types, including physical stress, is the differential regulation of the heat-shock genes. Among those is *rpoH*, encoding the heat-shock sigma factor σ^32^ ^26^. To test for possible induction of *rpoH* by mechanical stresses in colonies growing against external confinement, we used a laboratory strain of *E. coli* (JM105) expressing GFP under the control of the *rpoH* promoter, which was found to generate the same maximal pressure as its wild-type counterparts (Fig. 1c). Changes in GFP fluorescence per unit volume, obtained by normalizing the total fluorescence intensity measured with wide-field microscopy (WFM) to the volume of the chamber measured with CLSM at the same time point (Fig. 1e), were used to assess upregulation of *rpoH*. As long as the chamber roofs remained flat, the normalized fluorescence intensity values remained at the basal level, suggesting no detectable upregulation of *rpoH* throughout the course of colony expansion when the chambers were gradually filled with cells (Fig. 1d, e and Supplementary Fig. 5). A detectable increase in the normalized fluorescence, indicating increasing levels of *rpoH* expression, occurred at a time point when the roof was substantially deformed and the bacterial pressure on it reached ~0.75 psi (~5 kPa). As the roof became more deformed, the volume-normalized fluorescence increased by as much as 100% with respect to the level prior to the roof deformation. This *rpoH* expression increase during colony expansion was confirmed by measurements of fluorescence using both WFM analysis normalized to the bio-volume changes in the deforming chambers and by 3D reconstruction and individual plane analysis using CLSM (Fig. 1f and Supplementary Fig. 6), yielding consistent results. These results suggest that activation of the stress response machinery was concurrent with the onset of mechanical stress in the colony, as inferred from the roof deformation.

### Mechanical stress due to colony expansion leads to biofilm formation

**Fig. 2:**
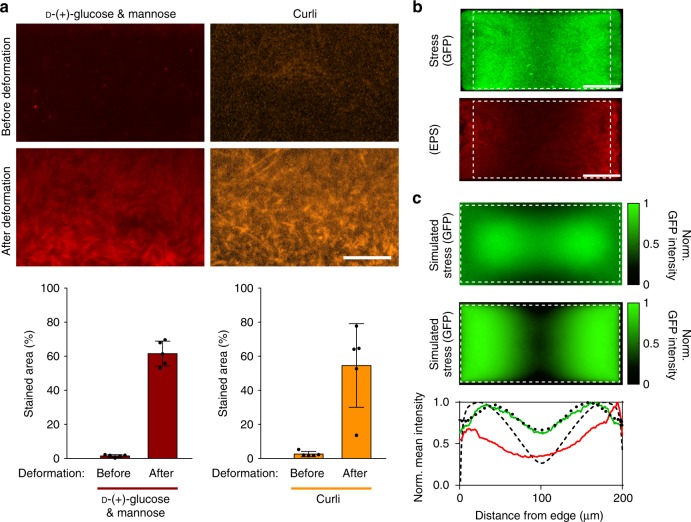
Spatial correlation between expression of biofilm-associated cell surface structures and biochemical stress response. **a** Expression of biofilm-associated cell-surface structures, EPS d-(+)-glucose and mannose groups (red) and curli (orange), as detected using rhodamine-labeled concanavalin A (10 µg/mL) and Congo Red dye (10 µg/mL), respectively, before (top row) and after (bottom row) roof deformation in uropathogenic *E. coli* populations. Scale bar, 10 µm. Bar graphs show major increases in expression of biofilm-related factors after roof deformation (*n* = 5). Error bars are SD. **b** Spatial distribution of GFP, as a reporter of *rpoH* expression, and of rhodamine-labeled concanavalin A (EPS stain) in *E. coli* JM105 after roof deformation. Dashed lines show boundaries of the deformable roof. Scale bars, 50 µm. **c** Agent-based (top) and mean field (bottom) simulations of the stress response distribution reproduce the characteristic patterns observed in GFP and EPS expression. The intensity of green color encodes the stress response. The spatial correlation between the experimentally measured expressions of GFP and EPS and simulated stress responses (agent-based: black dotted curve; mean field: black dashed curve) further supports the connection between mechanical stress and biofilm formation

Since *rpoH* upregulation has been linked with the onset of biofilm formation in *P. aeruginosa*^27^, we explored whether confinement-induced mechanical stress rendered uropathogenic *E. coli* (CFT073) colonies biofilm-like. We tested for the expression of known biofilm markers before and after chamber roof deformation, with a particular focus on factors involved in intercellular adhesion crucial for initiation of biofilm formation^28,29^. We examined two different types of such factors, exopolysaccharides (EPS)^11^ and curli^12^, using, respectively, rhodamine-labeled concanavalin A that specifically binds to d-( + )-glucose and mannose groups on EPS^30^, and Congo Red dye that has a high affinity to curli structures on the cell surface^31,32^. We compared the staining results for individual cells and cell clumps (Supplementary Fig. 7), bacteria-filled chambers with flat roofs (after colonies grew for 5–7 h, depending on chamber size and initial cell number), and bacteria-filled chambers with substantially deformed roofs (after ~5 h of growth past the point when the roof deformation first became visible; Fig. 2a). The results revealed a substantial increase in the expression of both biofilm markers after the roof became substantially deformed.

To further test for the direct effect of mechanical stress on the formation of biofilm-like structures, we transiently and rapidly increased the pressure in the pressure channel above the chambers, resulting in a transient invagination of the roofs. This invagination was imposed at the time when the colony has reached a fully packed (dense) state but has not yet developed the self-generated stress and initiated outward roof deformation. This initial state would mark a transition from a liquid-like property of the colony (allowing rapid relaxation of locally applied stresses throughout the colony leading to uniform stress distribution) to properties akin to jammed colloid states, where stress can remain local, leading to local stress response by the cells and ensuing formation of local biofilm-like structures. After removal of the inward pressure on the roof, the colony was allowed to grow to allow for maturation of biofilm-like features, which were localized in areas under the center of the membrane, where the invagination (and the pressure on cells resulting from it) was maximal (Supplementary Fig. 8a). During this time, the colony expansion led to cell reorganization and a more uniform self-induced stress distribution, leading to uniform *rpoH* induction. This uniform distribution is also common at the early stages of outward roof deformation from cell growth, where no inward deformation occurred (Supplementary Fig. 8b).

In contrast, continued growth of the colony until outward roof deformation (without initial roof invagination) resulted in a high spatial correlation between the two-dimensional distributions of *rpoH* upregulation and EPS expression within the bacterial colonies, which both displayed a characteristic butterfly shape (Fig. 2b and Supplementary Movie 4), further supporting the hypothesis that local stress triggers *rpoH* expression and transition to a biofilm-like phenotype. Interestingly, application of transient roof invagination coupled with growth until outward roof deformation resulted in the summation of the two staining profiles (Supplementary Fig. 9). There are two major sources of spatial non-uniformity in a growth chamber that can potentially lead to the butterfly pattern of the *rpoH* expression: the nutrient supply is through the two sidewalls adjacent to the flow-through channels; the vertical displacement of the roof is greatest near the middle of the chamber and zero near the side walls. We thus sought to explore the effect of these two spatial non-uniformities, using two simple computational models (see Methods for details). In the first model, spatially non-uniform nutrient supply was modeled in silico by maintaining nutrient levels constant at opposing chamber boundary zones to replicate supply by flow-through channels, and allowing diffusion and cellular consumption to define the nutrient levels in the rest of the chamber. Growth rate was assumed to be proportional to the local nutrient levels, and expression of *rpoH* was assumed to be proportional to the pressure and growth rate, because we hypothesized the mechanical stress to result from growth in a crowded and viscous extracellular environment. The roof deformation profile was simulated according to the plate-theory model^18, ^which was confirmed by our experimental measurement. Simulation of a mathematical model incorporating these assumptions indeed resulted in the butterfly-shaped growth-induced distribution of stress response essentially identical to that observed experimentally (Fig. 2c and Supplementary Movie 5). The model also predicted the formation of a shallow nutrient gradient from the supply sidewalls towards the interior of the chamber. The presence of such gradient was confirmed experimentally with a cAMP-CRP (cAMP receptor protein)-level reporting strain (Supplementary Fig. 10)^6,33^. The nutrient and growth rate gradients, in conjunction with the maximal vertical displacement of the roof at the middle, led to the bulk motion of cells from the sidewalls proximal to the flow-through channels into the interior chamber regions, where access to nutrients becomes even more restricted and cellular activity is expected to be reduced (Fig. 1b). This collective cell motion was also observed experimentally (Supplementary Movie 3). A second, mean field model that represented the process as a viscous fluid undergoing creep flow and incorporated identical assumptions yielded similar results (Fig. 2c). The agreement between the results of the simulations and experiments supports the model assumptions and provides further validation of the hypothesis that mechanical stress resulting from expansion of bacterial colonies in spatially confined regions can lead to formation of biofilm-like structures in uropathogenic *E. coli*.

### Antibiotic tolerance due to biofilm as a penetration barrier

**Fig. 3:**
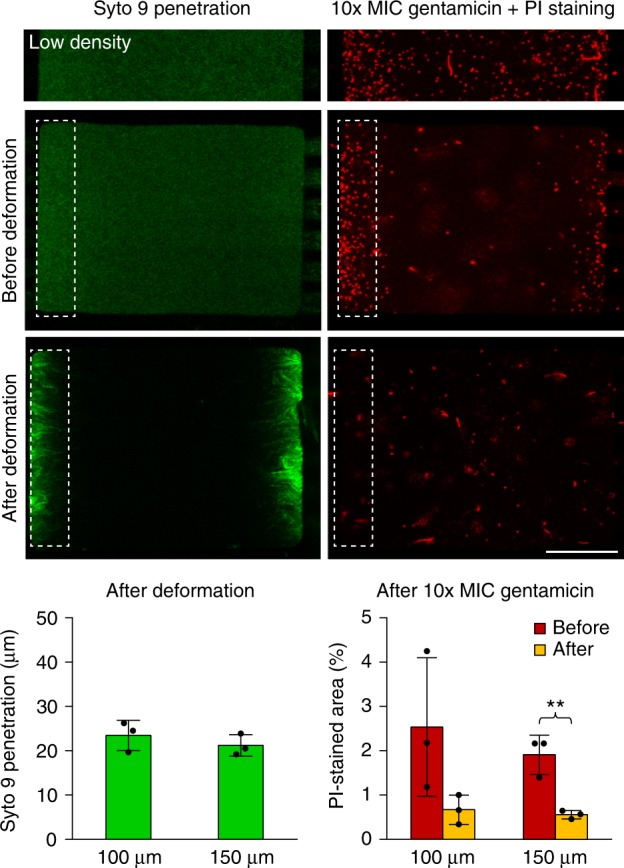
Confinement-dependent formation of a selective penetration barrier confers increased antibiotic tolerance. SYTO 9 (green, left) dye, mimicking positively-charged antibiotics was used to assess the penetrability of bacterial colonies at low cell density (top), high cell density before roof deformation (middle), and high density after deformation (bottom). Separately, antibiotic tolerance was assessed with 10x MIC gentamicin (15 µg/mL) treatment for 3 h followed by PI (red, right, to stain dead cells with compromised membranes) staining under identical density conditions. Dotted line boxes indicate regions proximal to the flow-through channels. Bar graphs indicate extent of SYTO 9 penetration after roof deformation and the percentage of PI-stained areas in regions proximal to the flow-through channels after antibiotic treatment in 100 and 150 µm wide chambers. Error bars are SD (*n* = 3); **P < 0.01; two-tailed Student’s *t*-test. Scale bar, 50 µm

A functional consequence of biofilm formation is increased antibiotic tolerance. Prior work indicated that the mechanisms suppressing the effectiveness of antibiotics in biofilms can be either adaptive or passive^34^. The adaptive mechanisms are characterized by activation of protective cellular machinery in individual cells, including the expression of degrading enzymes, activation of efflux pumps, and reduction of metabolism^35,36^. In the passive antibiotic resistance, EPS and other substances in the extracellular biofilm matrix shield cells from antibiotic compounds, which can have strong affinity to EPS or other biofilm-associated molecules^37^. For instance, whereas some antibiotics such as fluoroquinolones can readily penetrate the biofilm matrix^38^, positively-charged aminoglycosides are very ineffective in penetrating the negatively-charged EPS^39,40^. To test for the formation of a negatively-charged penetration barrier, constituting a passive mechanism, we used a DNA-binding, positively-charged fluorescent dye (SYTO 9) as a proxy for positively-charged antibiotics. We examined the penetration of SYTO 9 into uropathogenic *E. coli* (CFT073) colonies in the microfluidic device at a low density in the exponential growth phase, and at high densities prior to and subsequent to roof deformation (Fig. 3). In a parallel set of experiments, colonies in the microfluidic culture chambers were treated with the aminoglycoside antibiotic gentamicin for 3 h at a concentration 10 times greater than the minimal inhibitory concentration (MIC; as determined by an E-test outside the microfluidic device) under identical cell density conditions, followed by the counting of dead cells with compromised membranes using propidium iodide (PI). We observed that prior to dense packing, SYTO 9 uniformly penetrated the chamber, staining the cells throughout. Consistent with this result, the distribution of PI-stained dead cells following the antibiotic treatment was uniform throughout the chamber. However, when cells filled the chamber to capacity at high densities, but just prior to roof deformation, the antibiotic-induced cell death became more restricted to regions proximal to the capillary channels (in the areas containing more rapidly multiplying cells), although SYTO 9 penetration remained high. This suggests that reduction of growth rates in cells in the interior of the chamber confers a limited level of protection against antibiotic treatment, but fast-dividing cells adjacent to the side wall remain susceptible. After roof deformation, SYTO 9 penetration was significantly reduced, as was the cell death in the regions adjacent to the side wall, which is consistent with the emergence of the biofilm-mediated antibiotic penetration barrier. The extent of SYTO 9 penetration and antibiotic-mediated cell death did not depend on the chamber size, indicating that the penetration barrier was due to biochemical changes at the colony boundary (Fig. 3). In contrast to SYTO 9, a negatively-charged fluorescent dye that did not have affinity to extracellular structures (Alexa Fluor 488) uniformly penetrated bacterial colonies even after the roof became deformed (Supplementary Fig. 11). Overall, these results suggested that a hallmark of biofilm development—increased antibiotic tolerance—indeed becomes pronounced concurrently with biofilm marker expression within the chambers, revealing functional characteristics of the emerging biofilm-like structures.

### Biofilm formation due to self-generated stress within matrix-like environment

**Fig. 4:**
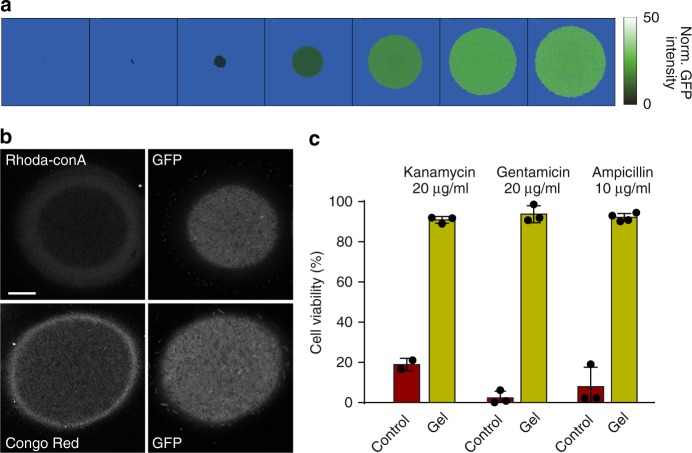
Biofilm growth in environment with mechanical resistance. **a** Filmstrip of a simulated colony growing in hydrogel. The green color scale encodes the level of stress response. **b** Spatial distributions of stress-reporting GFP and biofilm-like cell-surface structures in *E. coli* (JM105) colonies grown in 1% PuraMatrix hydrogel. CLSM was used to visualize both the rhodamine-labeled concanavalin A-stained EPS d-(+)-glucose and mannose groups and the Congo Red-stained curli after ~6 h of growth. Scale bar, 20 µm. **c** Viability comparison between planktonic and 6 h hydrogel cultures after exposure to 20 μg/mL of kanamycin (JM105) (*n* = 2 for control, *n* = 3 for gel), 20 μg/mL of gentamicin (CFT073) (*n* = 3), and 10 μg/mL of ampicillin (CFT073) (*n* = 3 for control, *n* = 4 for gel). Error bars are SD

Formation of biofilm-like colonies within the microfluidic culture chambers is a model of natural biofilm formation within small confined spaces, where the colony may not experience appreciable mechanical stresses until it fills the entire available space. However, in many circumstances, particularly during invasion and colonization of the host eukaryotic cells, expanding bacterial colonies can experience mechanical resistance from very early growth stages. Furthermore, the nutrient supply and mechanical resistance of the surrounding medium (e.g., the matrix of the host cell cytoskeleton) can be much more isotropic, leading to spherical shapes of biofilm-like colonies^41^. How does such a biofilm-like colony emerge? Can the mathematical model we developed and validated for the expansion of biofilm-like colonies in microfluidic chambers also inform our understanding of this process? To address these questions, we extended the computational model developed above by using a linear-elastic model to approximate the gradually increasing pressure resulting from continuous colony growth and expansion against the surrounding matrix (see Methods). The simulations indicated that initiation of *rpoH* expression can occur early in colony development, when the population size is small, as a result of pressure incurred from continuous expansion against the increasingly stressed surrounding matrix (Fig. 4a and Supplementary Movie 6). As the colony continued isotropic expansion, *rpoH* upregulation occurred at a higher level at the periphery, which was also the region of fastest cell growth due to unrestricted nutrient access. On the other hand, cell growth in the core of the colony declined due to decreased nutrient access, leading to reduced *rpoH* expression, but the increasingly sessile state of these cells also impeded the dilution of the existing, highly stable GFP proteins. As a result, the simulated colonies exhibited relatively spatially uniform levels of GFP in a sharp contrast to the steep spatial gradients of GFP in the butterfly-shaped patterns in the microfluidic culture chambers. The simulation results suggested that an isotropic and spatially uniform confining environment could make the increasingly sessile core cells express stress-related factors prior to their transition to dormancy, thereby causing stress response at all stages of colony development. Furthermore, if accounting for the putative threshold-like dependence of the biofilm development on the stress response intensity, the model suggested that the biofilm markers would be expressed at the periphery of the colony, after the colony reaches a sufficient size. Given the mutual positive cross-regulation between the local proliferation and stress-induced biofilm formation, the biofilm development was expected to be sudden, in the form of a shell encasing the colony.

We experimentally tested the model predictions by embedding JM105 *E. coli* cells reporting *rpoH* expression into 1% PuraMatrix peptide hydrogel matrix, which facilitated the generation of coherent colonies (Supplementary Fig. 12), to monitor the distribution of stress during colony expansion. While the bulk material properties of this gel^42–44^ are different from those of PDMS^45–47^, both materials are expected to exert considerable resistance to deformation and elicit cellular responses. In addition to isotropic and uniform permeation of nutrients into the colony, growth in gel matrix allowed the colony to experience mechanical resistance to expansion from the very beginning, which might better approximate formation of biofilm-like structures in host epithelial cells. After ~6 h of growth following the initial seeding, colonies of different sizes were found in highly-packed states, indicating the ability to maintain cohesiveness within the 3-D environment (Fig. 4b). In contrast to the butterfly-shaped *rpoH* expression pattern observed in the microfluidic culture chambers, the *rpoH* expression pattern in the hydrogel colonies revealed by CLSM was, as predicted by the model, relatively uniform, with indistinguishable levels of GFP between the core and periphery of the colony (Fig. 4b and Supplementary Fig. 13). Staining with rhodamine-labeled concanavalin A and Congo Red in well-formed colonies resulted in detectable and near uniform fluorescence in internal regions of the colonies, indicating the expression of EPS and curli throughout the colonies and the ability of these stains to penetrate into the colonies (Fig. 4b and Supplementary Figs. 13 and 14). There were also much brighter halos of staining at the periphery of the colonies, indicating elevated levels of biofilm markers EPS and curli secreted at the periphery, again in agreement with model predictions (Fig. 4b).

To verify the effectiveness of these biofilm-like colonies to form a barrier to antibiotics in an isotropic growth setting, JM105 colonies grown for 6 h in unconstrained culture medium and in hydrogels were subjected to 20 μg/mL of kanamycin insult. The results indicated a significant increase in viability in hydrogel, even if the colonies were relatively small (Fig. 4c and Supplementary Fig. 15). These small colonies also had a high level of *rpoH* expression, suggesting that colonies in hydrogel were exposed to self-induced stress starting from early stages of expansion and that this stress led to enhanced antibiotic resistance. This experiment also provided strong evidence against the nutrient access limitation as a likely trigger of biofilm emergence and antibiotic resistance enhancement. In addition, uropathogenic *E. coli* colonies grown for at least 6 h in the hydrogel and subsequently subjected to 20 µg/mL gentamicin or 10 µg/mL ampicillin insult displayed a significant increase in viability as compared to the same cells planktonically grown in bulk culture. This experiment provided further evidence that growth under mechanical stress can confer substantial protection against certain antibiotics (Fig. 4c and Supplementary Fig. 16). Taken together, the experimental results demonstrate that expression of stress-dependent proteins, caused by mechanical resistance of the environment and occurring throughout the course of colony growth, is just as effective as a sudden, abrupt stress onset at eliciting peripheral biofilm production and conferring the accompanying antibiotics tolerance.

## Discussion

Mechanical environment is increasingly seen as an important determinant of function in a variety of cell and tissue types^48,49^. While stresses stemming from the external environment have been implicated in the formation of biofilm-like structures^50^, we found that this important defensive strategy of *E. coli* is temporally and spatially correlated with the onset of self-imposed physical stress (evidenced by the emergence of a biochemical stress response) as a result of colony expansion in confining environments. This result strongly suggests that the biofilm development could be a direct consequence of evolving mechanical stresses, a common feature of the complex natural micro-environments. Furthermore, the gradual onset of physical stress in a hydrogel environment made the colony express stress-dependent factors at all stages of growth, priming the colony to react much quicker and with increased sensitivity to environmental changes. The temporal and spatial discrepancy in the distributions of stress and biofilm markers under these conditions, which mimic intracellular milieu, suggests that biofilm formation requires exceeding a threshold of the stress levels, which could depend on colony size, nutrient availability, degree of confinement, and the mechanical properties of the confining environment. Overall, these results suggest that physical confinement of an expanding colony, e.g., within a host cell, and mechanical resistance of the environment to the colony growth can be important factors in triggering the biofilm development.

Our results showed that the stress buildup was spatially correlated with increased cell proliferation, which in turn occurred in the regions of abundant nutrient access. Why would these regions accumulate higher physical and thus biological stress levels? The increasingly dense cell colony could have rapidly rising effective viscosity, suggesting that local mechanical stresses might take progressively longer to relax (this is in contrast to growth in aqueous environment, where the stress relaxation is effectively immediate). Therefore, in regions of high cell proliferation, the rate of the buildup of the cell division-induced mechanical stress can exceed the rate of its relaxation, leading to a progressive increase in the stress levels. Even for a relatively low viscosity (*μ*) on the order of 1 kPa•s (while 10^6^ times greater than water, still much lower in comparison to many tightly packed bacterial communities found in nature^51^), the viscous force per unit area experienced by a bacterial cell with a doubling time (*τ*) of 25 min would be on the order of 3 *μ*/2*τ* = 1 Pa from Stokes’ law^52^, which is on the high end of physiologically relevant viscous forces^50,53,54^. Our findings and previous studies^55–58^ suggest that bacteria can respond to changes in the viscous forces by alteration of gene regulation, ultimately leading to EPS expression and biofilm formation, which may further affect the mechanical properties of the cell micro-environment. Hence, the onset and progression of the biofilm formation can be subject to a positive feedback and thus occur in a switch-like manner, provided that the local stress levels exceed a threshold value. This positive feedback, however, can be counter-balanced by a negative feedback between the stress buildup and the rate of colony expansion, due to transition to slower growth phenotypes, and possibly higher cell mortality^41^. The interplay between these feedback interactions and sensitivity of cells in biofilms to other aspects of local environment, such as availability of nutrients and presence of antibiotics, can potentially lead to diverse and highly adaptive biofilm growth allowing considerable structural complexity.

Overall, our study suggests that formation of biofilm-like structures can strongly depend on self-organizing mechanisms resulting in the interplay between EPS secretion and continued colony growth. It can be triggered by the initial growth in confining spaces, which has been implicated to be actively sought out by starved, motile cells. The buildup of biofilm-like structures can thus be a part of the adaptive defensive strategy of bacterial colonies, providing protection from adverse environmental stresses and antibiotic treatment. The increasing concerns about antibiotic tolerance, and growing use of biocompatible medical devices and tissue engineering applications make the understanding of biofilm development ever more important. Our study both provides the platform for controlled biofilm analysis and reveals new mechanisms underlying biofilm formation.

## Methods

### Bacterial strains and growth conditions

*E. coli* strain JM105, with low copy number plasmids (pCS101 origin, ampicillin resistance with GFPuv gene, *gfp*_*uv*_) expressing GFP under the control of the heat-shock transcription factor σ^32^ promoter, was used to monitor the upregulation of stress response genes in the bacterial colonies. Unless stated otherwise, antibiotic resistance experiments were performed with CFT073 *E. coli*, an uropathogenic strain involved in murine urinary tract infections. JM105 and CFT073 were generous gifts from the Bentley group (University of Maryland, College Park, MD) and the Donnenberg group (University of Maryland School of Medicine, Baltimore, MD), respectively. *S. cohnii* and *P. aeruginosa* strains were obtained from ATCC.

Prior to all experiments, bacterial cells were inoculated from a single agar plate colony and were grown at 30 °C in low salt (4 g/L) LB medium supplemented with antibiotics, where appropriate, overnight. Overnight cultures were diluted 1:100 the next day into fresh medium and grown at 30 °C to mid-log phase (0.2–0.3 OD_600_) before being used for experiments.

### Device design, fabrication, and operation

The device consists of two layers: a culturing layer on the bottom and a channel layer on top. The layout of the bottom layer is similar to that of the microfluidic chemostat^17^, consisting of an array of 8 parallel flow-through channels, each with a depth of 6 μm, which are continuously perfused with fresh media from the inlets. 6 μm deep chambers reside in between the flow-through channels and are connected to the flow-through channels by 0.65 μm deep capillaries. The dimensions of the chamber used are 200 μm in length and either 50, 100, 150 or 200 μm in width. The top layer consists of an array of channels that run on top of the culture chambers, resulting in the thin, deformable layer of PDMS membrane between the layers that serves as the roof of the culture chamber.

The device was fabricated using multiple-layer soft lithography, with the silicon master molds for each layer fabricated using standard photolithography techniques^17^. PDMS (Sylgard 184, Dow corning, Midland, MI) with 5:1 and 20:1 mixtures of resin and catalyst were used for the top and bottom layer, respectively. The top layer was partially cured before individual devices were cut and holes were bored to create inlet and outlet ports. The top layer pieces were then aligned and bonded to the partially cured bottom layer in the oven to complete the curing process. The final devices were immersed in a 0.01 N HCl solution for 1 h at 80 °C, baked for at least 30 min in a 150 °C oven to increase hardness and Young’s modulus, hermetically sealed to #1.5 microscope cover glasses, and further baked at 80 °C overnight.

Prior to all experiments, the device was filled with 1% BSA in PBS solution to prevent attachment of cells to the cover glass. The BSA solution was replaced with LB medium supplemented with antibiotics, where appropriate, before cells were loaded and flow was driven as described in the previous study^17^.

### Device calibration and pressure measurement

For calibration, the chip was perfused with fluorescent dyes in both layers (Alexa Fluor 555 on top and Alexa Fluor 488 on the bottom) and external hydrostatic pressure was systematically modulated from 0 to 3 psi and the corresponding vertical displacements of the PDMS membrane were measured in the cross-sectional view of the chamber captured by confocal microscopy. Displacement measurements of the PDMS membrane were performed with multiple chambers of different sizes for each chip via 3-D reconstruction of confocal image stack. The same calibrated chambers were then used for pressure measurements of bacterial colony growth.

### Cell staining

The following reagents were used in our study: (1) Rhodamine-labeled Concanavalin A (10 μg/mL; RL-1002, Vector Laboratories, Burlingame, CA), (2) Congo Red (10 μg/mL; C6277, Sigma-Aldrich, St. Louis, MO), (3) Propidium iodide (10 μg/mL; P4864, Sigma-Aldrich), and (4) SYTO 9 Green fluorescent nucleic acid stain (S-34854, Thermo Fisher Scientific, Waltham, MA). Prior to each staining procedure, LB medium was removed from the device with a PBS wash. The appropriate staining solution was added and allowed to perfuse the device for 30 min in the dark, followed by another PBS wash prior to imaging. For staining of samples in hydrogel matrices, existing media was removed and 4% paraformaldehyde fixative was added for 20 min prior to the staining.

### Microscopy and data analysis

Epi-fluorescence and phase contrast images were captured with a 40 × /0.75 Plan-fluor dry objective and a monochrome Cascade 1 K digital camera (Photometrics, Tucson, AZ) on a Nikon Eclipse TE2000 inverted microscope equipped with a computer-controlled motorized stage. Colonies in the selected chambers were imaged every 10 to 15 min using Slidebook (3I, Denver, CO). To capture 3-D time lapse images of microcolony growth, confocal laser scanning microscopy was performed with a 40 × /1.3 Plan-Neo oil objective on a Zeiss LSM 510 Meta confocal microscope. A fast scan was performed to determine the top and bottom of the microchambers prior to performing a full resolution scan. Projected 3-D images and vertical cross-sections were generated with the LSM image browser software. The excitation/emission wavelengths were 395-425/510 nm for GFP_uv_, 485/498 nm for SYTO9, 535/617 nm for propidium iodide, 633/650 nm for Congo Red, and 514/625 nm for rhodamine-labeled concanavalin A.

The dynamics of the stress response onset was calculated by taking the average of the *rpoH*-GFP expression mediated fluorescence intensity data within the region of interest, and normalizing it to the initial time point. In the analysis of the epi-fluorescence data, the results were also normalized to the volume of the cell-filled deformed chamber. To perform this normalization, we confirmed that the elastic plate-theory can be used for the membrane deformation analysis, as described below. We used the 3D volume reconstruction (Supplementary Fig. 6) from confocal imaging to validate the following formula:

1 $$V = V_0 + \mathop {\int }\limits_0^{z_{{\mathrm{max}}}} \mathop {\int }\limits_0^b \mathop {\int }\limits_0^a z\left( {x,y} \right){\mathrm{d}}x{\mathrm{d}}y{\mathrm{d}}z$$

2 $$z(x,y) = \frac{{z_{{\mathrm{max}}}}}{4}\left( {1 - {\mathrm{cos}}\frac{{2\pi x}}{a}} \right)\left( {1 - {\mathrm{cos}}\frac{{2\pi y}}{b}} \right)$$

These formulas represent the first approximation for the deflection of a rectangular plate of length *a* and width *b* with all sides fixed^59^. The values of *x* and *y* here represent the coordinates corresponding to the length and width dimensions respectively, while the coordinate *z* is orthogonal to *x* and *y* and oriented in the direction of the deflection. This deflection adds an additional volume to the initial volume *V*_0_, resulting in the estimate that can be obtained from the measurement of *z*_max_. The same theory also predicts that the displacement of the *z* coordinate itself is linearly dependent on the applied pressure, as follows^18^

3 $$z_{{\mathrm{max}}} = \alpha \frac{p}{E}\frac{{a^2b^2}}{{t^3}},$$

where *t* is the membrane thickness, *a* and *b* are as defined above, and *E* is the modulus of elasticity. For a given membrane the coefficient *α* is an empirically determined value that depends on aspect geometry, mechanical properties of the material and edge constraint.

To address the question of theoretically predicted pressure distribution within the chamber, we used the formula above as well as performed simulations (in ANSYS 10.0 simulation environment), for the following experimental conditions: *t* = 20 μm, *a* = 150 μm and *b* = 160 μm; *E* = 0.35 MPa^45^ (data from one of the experimental chamber geometries, see an example in Supplementary Fig. 2). These results were consistent with the calibration data in Supplementary Fig. 1 and the simulations using the same theory as implemented in ANSYS for different chamber widths. We independently determined the change of the deflection for various chambers as a function of time of the bacterial colony expansions (Supplementary Fig. 4). The result was fully consistent with the observations of the calibration experiments (Supplementary Fig. 1c) This allowed us to estimate the added volume as a function of time, using it to normalize the epi-fluorescence measurements with the *rpoH* stress reporter. As indicated above, we validated the results by comparison at selected time points to the 3D reconstruction from confocal imaging, see an example below in Supplementary Fig. 6.

We also noted that, due to a quick equilibration of *rpoH* expression, on a time scale that is much faster than the experimental time scale (see Fig. 1, also illustrated in a more convenient fashion in Supplementary Fig. 5), we can assume that the concentration of the corresponding sigma quickly reaches a steady state, as described by:

4 $$\frac{{\mathrm{d}}c}{{\mathrm{d}}t} = f - k_{\mathrm{d}}c = 0,$$

where *f* is the rate of production, *c* is the concentration of the sigma factor and *k*_d_ is the rate of dilution due to continuous cell division, yielding the rate of change of 0. Thus, it is the concentration *c* rather than its rate of change that is directly related to the production function *f*:

5 $$c = \frac{f}{{k_{\mathrm{d}}}}$$

Therefore, we assumed that, due to the continued rapid cell division following the initiation of chamber deformation, the analysis of the GFP fluorescence intensity levels (rather than the time derivative of this intensity) is adequate for representing the dynamics of *rpoH* expression. Stained areas are calculated by dividing the number of pixels that have intensities higher than a threshold intensity determined by Otsu’s method, and dividing it by the total number of pixels within the region of interest.

### Preparation of hydrogel matrices

To prepare the hydrogel-grown colonies, overnight LB cultures were diluted 1:100 and grown to mid-log phase as before. Cells were collected by low speed centrifugation and washed twice with sterile 0.85% NaCl solution. The cells were mixed with 1% PuraMatrix Peptide Hydrogel (Corning Life Sciences, Teterboro, NJ) that has been sonicated for 30 min to reduce the viscosity. Addition of medium induces changes in salt concentrations necessary for rapid gelation to occur, thereby encapsulating the cells within the hydrogel. Under physiological conditions, PuraMatrix Peptide Hydrogel forms a transparent hydrogel with an average pore size of 50–200 nm. The LB medium was changed 5 min after gelation occurred to equilibrate the pH, and every 3 h thereafter.

### Modeling of colony growth inside the chambers and hydrogel

The butterfly shape of the stress response distribution in the colonies confined in microfluidic devices can potentially be explained by two contributing factors: non-uniform nutrient access and anisotropic membrane deformation profile. Cells grow faster on the sides of the chambers closest to the micro-slits connecting them to flow-through channels due to better access to nutrients. Faster cell growth can lead to a faster stress buildup, hence higher GFP/EPS expression. Similar to a creeping-flow in a pipe, the growing cells are pushed towards the interior into the areas of the deforming membrane (the chamber roof), where resistance to flow is lower due to a relatively higher membrane compliance, resulting in the final stress profile. This explanation is corroborated by two different implementations of simulated growth-induced mechanical stress (Fig. 2c) in a chamber with dimensions similar to those used in the experiment, as described below. It is also supported by the 3D stress distribution shown in Supplementary Movie 4 and visualization of creeping-flow-like cell displacement seen in Supplementary Movie 3.

The agent-based simulation, performed using NetLogo^60^, models the individual cells as discrete entities, within a 2-D grid, and simulates their behavior under non-uniform nutrient conditions, and within an anisotropically-compliant roofed chamber. Collective behavior emerges from the interaction between individual cells, which are governed by a set of rules. Cells were modeled as circles with a diameter of *D* = 1, and the chamber dimensions were set relative to the cell diameter, with *H*_*x*_ (chamber length) = 140D, and *H*_*y*_ (chamber width) = 70D. Since the model cells are circular instead of rod-shaped, the dimensions of the chamber in the simulation were set such that a similar aspect ratio as well as cell number were maintained as compared to the experiment. The perimeter of the chamber is defined by a separate set of agents, which prevent the movement of cells beyond the confines of the chamber. Any movement, such as the expansion of the colony due to growth, that result in an overlap between a cell and a wall agent will result in a change in the heading of the cell such that it will face away from the wall. The same mechanism also prevents the overlapping of cells with other cells, which can occur if the cell occupancy exceeds the cell capacity for the given location, by aggregating the relative headings of all the overlapping cells and assigning new headings which minimize overlapping, thereby allowing the colony to expand to fill the chamber prior to its full occupancy. Once 99.95% of the simulated chamber area is occupied with cells as a result of the population growth and expansion within the chamber, a buildup of pressure occurs, which is proportional to the average occupancy of the chamber (total number of cells/area) normalized to the basal capacity. The increase in pressure results in the simulated deformation of the membrane, which is mimicked by an increase in the cell capacity of a given space where a cell resides. The extent of membrane deformation is proportional to the pressure in the chamber, and is approximated by increasing the capacity for cell occupancy at each point in the chamber according to the profile defined by Eq. (2), as dictated by the plate-theory model^18^ (see the discussion of validation of the model above). Negative differences in capacity and occupancy, as well as the amount of overlap with neighboring cells, lead to the mutual repulsion of inherently non-motile cells, resulting in bulk movement of growing cells toward the interior of the chamber as the roof is progressively deformed.

The model achieves non-uniform growth rate by maintaining a constant nutrient level of 10 at two opposing edges of the chamber, allowing a gradient of nutrients to form as a result of diffusion and cellular consumption. Diffusion is modeled as an equal distribution of nutrient shared to the neighboring regions, whereas maximal consumption rate is set to 0.008. The nutrient consumption rate of individual cells is attenuated by increases in the pressure, and is also proportional to the local level of nutrients relative to the source. Nutrient consumption results in an equal loss of nutrient in the local space. Cellular division occurs when a pre-defined level of nutrient (15) has been consumed. When a cell divides, a new cell is generated and the nutrient level needed for division for each cell is reset to half of the original cell, with a 5% variation added for randomness. Therefore, a gradient of growth rates emerges from the nutrient gradient within the chamber, with higher rates of growth closer to the source of nutrients due to faster consumption, and a faster rate of reaching the threshold necessary for cell division. Stress, indicated by GFP production and accumulation, is assumed to positively correlate with the pressure, as well as nutrient consumption rate when pressure is positive. Cellular escape at the two sides of the chamber was modeled with a Monte Carlo simulation. The final stress profile was obtained by integrating the stress in the z-direction within a defined radius of the cell to enable smoothing. Each time step in the simulation of the model includes simulation of the diffusion of nutrients, cell capacity change and pressure change of the environment, followed by the simulation of the consumption of nutrients, division of cells, movement as a result of overlap, and stress profile for each individual cell. Cellular escape from the chamber is simulated at the end.

The hydrogel model is essentially identical to the model above, following the same rules, but with a few key differences. There is no preset boundary which physically limits the expansion of the colony like in the case of chamber growth. Without a boundary, the expansion of the colony depends only on its ability to consume nutrients and divide. The growth remains negatively affected by a buildup of pressure, but pressure experienced by colony as a result of expansion within the isotropic hydrogel matrix is now modeled as a compressed spring using Hooke’s law. The pressure is set to be proportional to a factor representing the stiffness of the hydrogel material (0.2), as well as the difference between the colony radius at time = t and the colony radius at time = 0, which, in this case, was the radius of a single cell (0.5 D).

The mean field simulation assumes that the chamber is filled with a viscous fluid that undergoes creep flow. The non-uniform growth rate is modeled by a gradient of rates at which fluid enters the chamber from the bottom, so that there is maximum growth at the sides and less growth at the center. The membrane deformation is mimicked by an equivalent pressure-release profile on the top of the chamber. Stress is assumed to be directly proportional to the growth rate (i.e., the creep rate). The final stress profile (Fig. 2c) was obtained by integrating the stress in the *z*-direction, which shows good agreement with the measured GFP and EPS expression profiles. The simulation was performed using the COMSOL laminar flow module, which uses by default the compressible-flow formulation of the Navier-Stokes equations. The fluid density was set to 10^3^ kg/m^3^, and the viscosity to 10^5^ Pa∙s. The dimensions of the chamber used in the simulation were *H*_*x*_ (chamber length) = 200 μm, *H*_*y*_ (chamber width) = 100 μm, and *H*_*z*_ (chamber height) = 6 μm. The walls had no-slip boundary conditions. The bottom surface of the chamber was set as an inlet with a normal inflow velocity proportional to

6 $$v = 1.5 + {\mathrm{cos}}\frac{{2\pi x}}{{H_x}},$$

to establish the gradient mimicking growth rate. The top surface of the chamber was set as an outlet with an exit pressure proportional to Eq. (2) to mimic fluid flow due to membrane deformation. To calculate the stress, the magnitude of the flow velocity was integrated in the vertical (*z*) direction.

### Statistical analysis

At least three independent tests were done for each experiment. *F*-test was performed to determine variances between samples. 2-tailed *t*-tests were used for comparisons.

### Code availability

The codes used in this study are available from the corresponding author upon request.

## Electronic supplementary material

Supplementary Information

Description of Additional Supplementary Files

Supplementary Movie 1

Supplementary Movie 2

Supplementary Movie 3

Supplementary Movie 4

Supplementary Movie 5

Supplementary Movie 6

## Data Availability

The data that support the findings of this study are available from the corresponding author upon request.
