# Supplementary material for: Self-induced mechanical stress can trigger biofilm formation in uropathogenic Escherichia coli
Source: Nat Commun. 2018 Oct 5;9:4087. doi: 10.1038/s41467-018-06552-z (PMC6173693; doi:10.1038/s41467-018-06552-z)
Supplement: Supplementary file 1 — Supplementary Information [file 41467_2018_6552_MOESM1_ESM.pdf]

Self-induced mechanical stress can trigger biofilm formation in uropathogenic *Escherichia coli*

Chu et al.

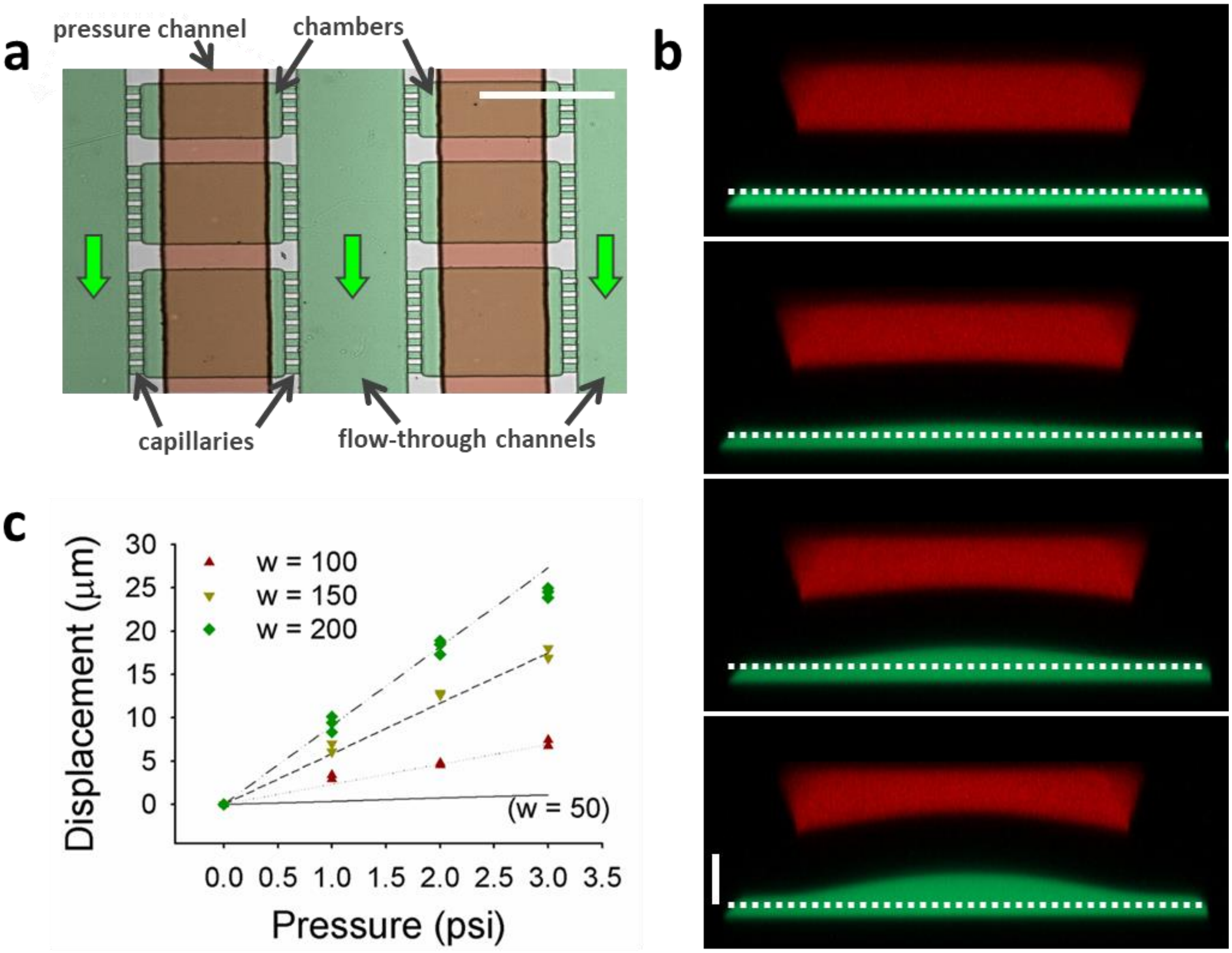

Supplementary Figure 1. Microfluidic device calibration. (a) A micrograph of a portion of the chamber array in the microfluidics device. The chamber and flow-through channel layer has been highlighted in green, while the overlaying channel layer used to create the deformable chamber roof has been highlighted in red. Scale bar, 200 µm. **(b)** The deformation of a chamber roof increases as progressively elevated uniform external pressure is applied to the chamber layer during calibration, as visualized with 3-D reconstruction from confocal imaging, using solutions of fluorescent dyes Alexa Fluor 488 (green; chamber layer) and Alexa Fluor 555 (red; pressure channel). Scale bar, 20 µm. **(c)** A representative calibration plot is obtained by plotting the displacement of the thin membrane as a function of the applied external hydrostatic pressure. The lines super-imposed onto the data points are obtained from the analytic

and simulation analysis of the corresponding thin plate deflection theory, described in the Methods section.

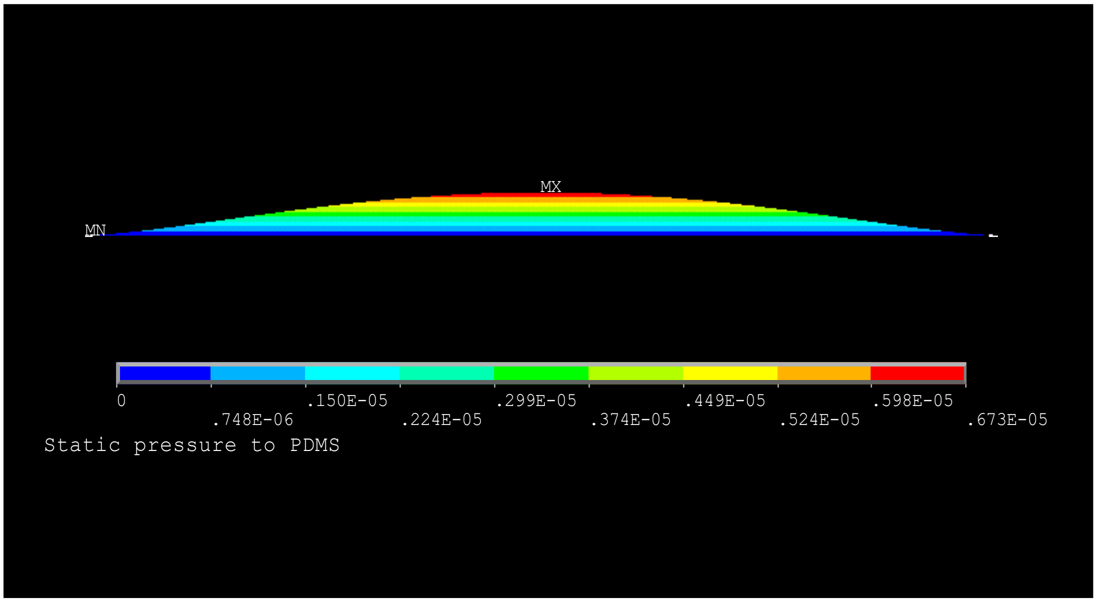

Supplementary Figure 2. Simulation of membrane deformation and stress profile. A numerical simulation of the deformation of a membrane that is 20 µm thick, 150 µm wide and 160 µm tall (long), and has the elastic modulus (corresponding to that of PDMS) of E = 0.35MPa, under 1 psi pressure. The resulting membrane deformation (deflection) is approximately 5 µm. The normalized stress values are color coded. The results are fully consistent with the predictions of the plate deformation theory provided in the text. The simulation was performed in ANSYS 10.0.

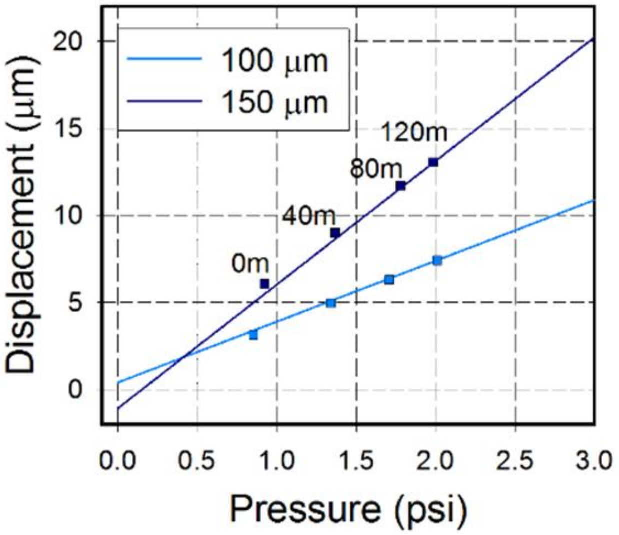

Supplementary Figure 3. Pressure dynamics as a result of confined growth. The dynamics of pressure increase generated by expanding *E. coli* colonies confined in chambers of different widths. The values are estimated from the corresponding deformation of PDMS membranes forming the roofs of the microchambers. Pressure values were determined from the displacements of the membrane in z-direction at time points 0 min, 40 min, 80 min, 120 min of growth after the chamber was filled with *E.coli* cells. The calibration procedure is described in the text and shown in Supplementary Fig. 1c. The results were independently validated by confocal imaging experiments at randomly selected time points, as shown in Supplementary Fig. 6. The measurements were performed in 100 μm-and 150 μm -wide chambers.

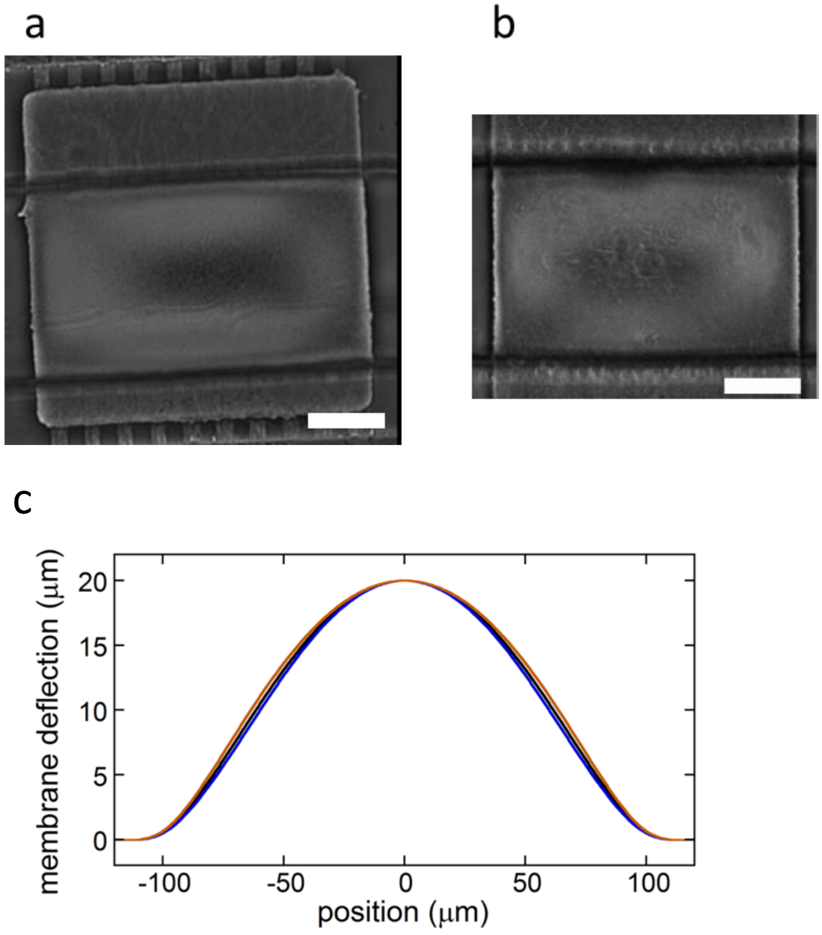

Supplementary Figure 4. Comparison of chambers with pressure channel alignment offset. (a,b) Phase contrast images of **(a)** a chamber with a shift in the alignment of the pressure channel above it, and **(b)** a chamber with the symmetrically aligned pressure channel. Note the 'lens effect' of the deforming roof of the expanding chamber, indicting the area of the maximum membrane deflection at the centers of the regions of the overlap of the pressure channel and the chamber. Scale bars, 40 μm. **(c)** Simulations of the deformation of a 15 μm thick membrane on top of a 200x200 μm, 6 μm deep chamber, with various pressure distributions applied. We considered 3 different spatial distributions of the pressure: an even distribution over the entire 200x200 μm square (black curve), 2x greater pressure on the central 100x100 μm square than on the rest of the membrane (red curve), and 2x smaller pressure

on the central 100x100 µm square than on the rest of the membrane (blue curve). The actual values of the pressure were normalized to achieve the same membrane deformation of 20 µm in the middle.

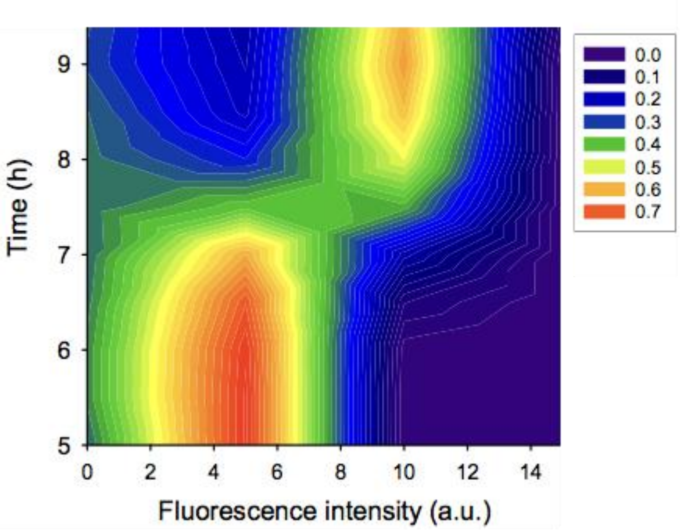

Supplementary Figure 5. Stress response dynamics in an expanding colony. The data from an experiment similar to the one shown in Fig. 1d, with the re-normalized range of the arbitrary units for the fluorescence intensity values and a different window of measurement with respect to the onset of the chamber deformation (around 7 hrs. on this example). The data were converted into the contour plot by interpolating and smoothing across individual histograms. The results suggest a rapid onset and equilibration with a new steady fluorescence response level.

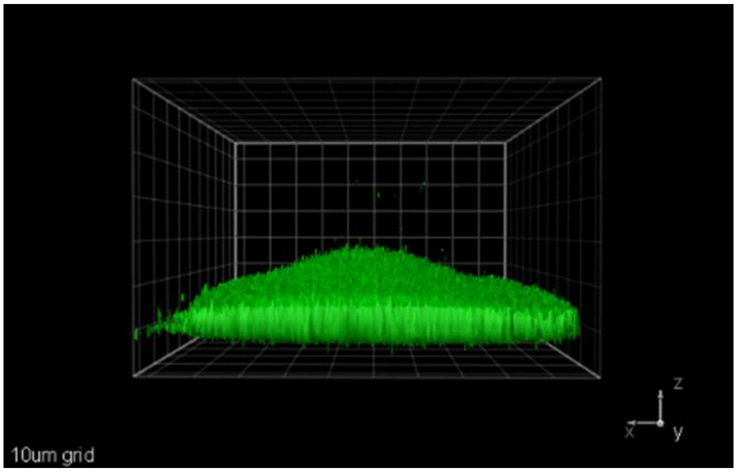

Supplementary Figure 6. 3D reconstruction of colony. A 3D reconstruction of a confocal z-stack of an *E. coli* colony expressing GFP under the *rpoH* promoter control within a fully deformed chamber.

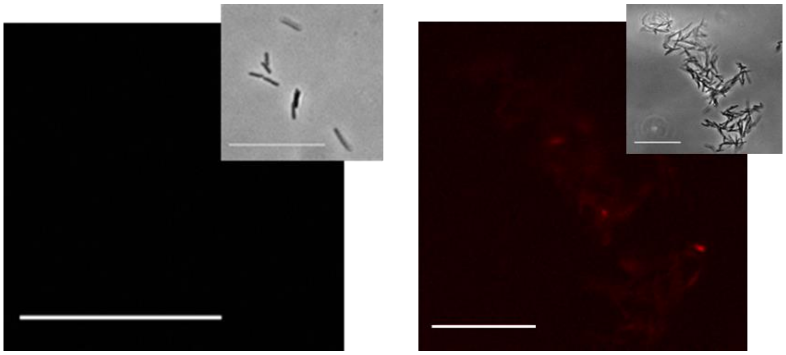

Supplementary Figure 7. EPS staining in sparse cells. Epi-fluorescence imaging shows exopolyssacharide (EPS) expression in individual cells (left) or cell clumps (right), as indicated by rhodamine-labeled concanavalin A stain. Scale bar, 10 µm

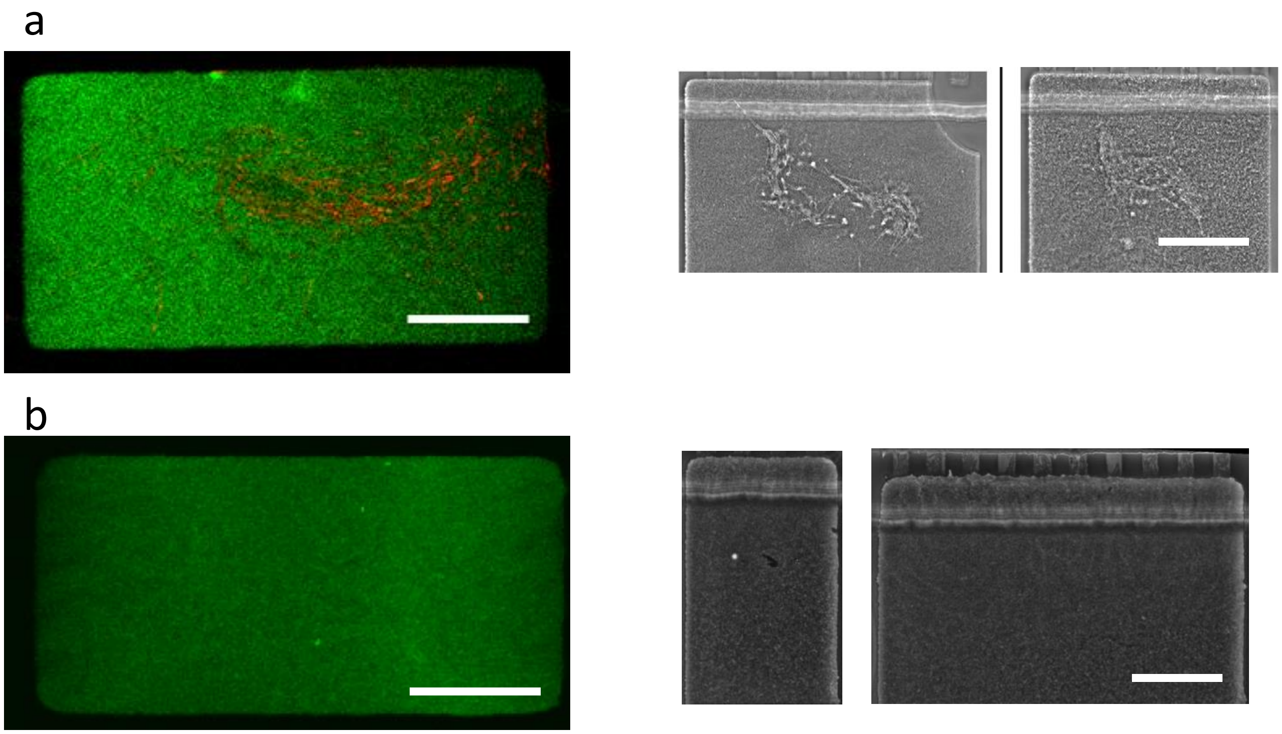

Supplementary Figure 8. Direct application of pressure. (a) Examples of GFP (under the control of the *rpoH* promoter) and rhodamine-labeled concanavalin A staining, and solid structure growth in growth chambers following their partial expansion with a transient inward membrane (chamber roof) deformation. The inward deformation was performed by increasing the pressure in the pressure channel to approximately 1 psi at the point of initiation of the outward deformation of the chamber roof, performed for 15 min. This was the time point immediately following the filling of the chamber with the expanding *E. coli* colony. The staining was performed after an additional hour of colony expansion following the inward deformation. The central region, where the staining is concentrated as visualized using confocal imaging, is the area of the highest inward membrane deformation, expected to lead to the highest amount of stress onto the cells. The even distribution of GFP is indicative of the additional input of the self-induced stress during the one hour of outward colony expansion. **(b)** The early stages of outward roof deformation as a result of cell growth, without initial roof invagination, also results in uniform GFP expression. Scale bars, 50 µm.

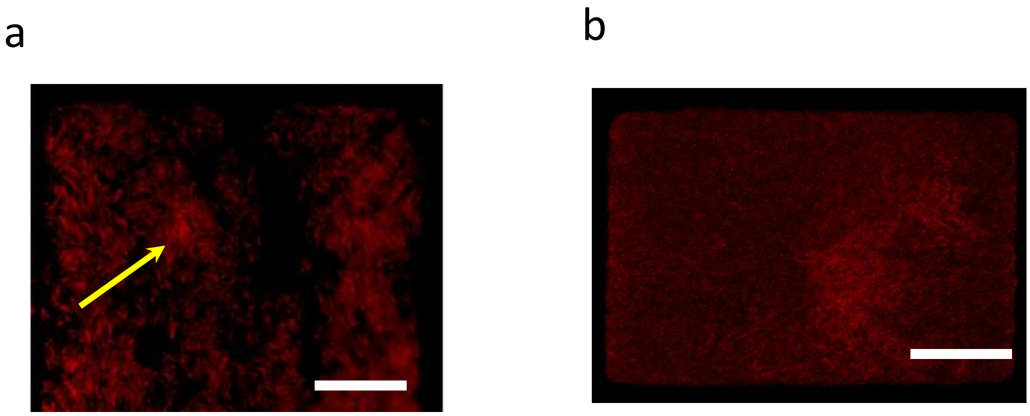

Supplementary Figure 9. EPS staining after direct pressure application. (a) An example of a confocal image using rhodamine-labeled concanavalin A stain in a 150 µm -wide chamber following its complete expansion after a transient inward membrane (chamber roof) deformation. Note both the expected ‘butterfly’ pattern and the unusual increased staining at the central region, indicated by the arrow. The central region is the area of the highest inward membrane deformation, expected to lead to the highest amount of stress onto the cells. **(b)** Control stain with rhodamine-labeled concanavalin A at the time when the colony has reached a fully packed (dense) state, but has not yet developed the self-generated stress and initiated outward roof deformation, demonstrated minimal staining as well as the lack of localization. Scale bars, 50 µm.

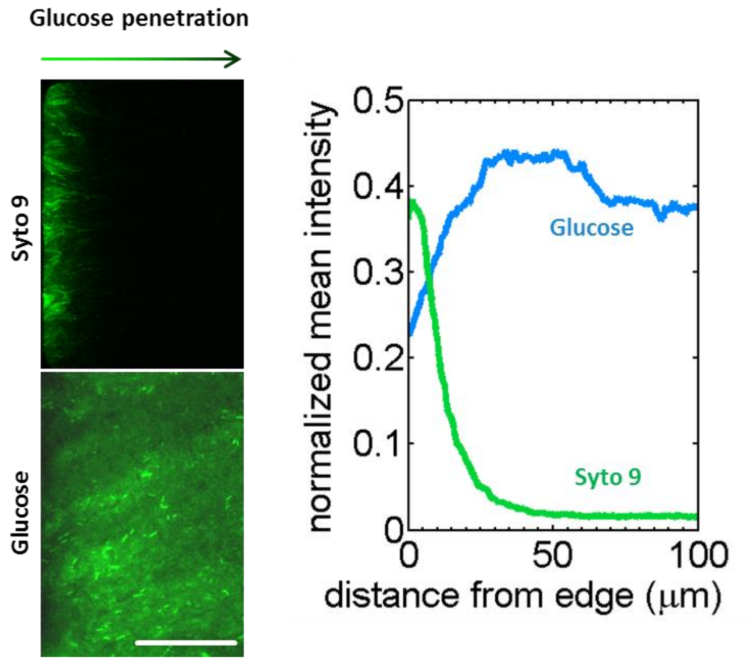

Supplementary Figure 10. Nutrient gradient formation within filled chamber. Detection of a nutrient gradient from the supply towards the interior of the chamber, with a cAMP-CRP (cAMP receptor protein) level reporting strain expressing at low levels in regions proximal to the exits, and increasing in expression towards the interior of the chamber until a maximal level is reached. Scale bar, 50 μm.

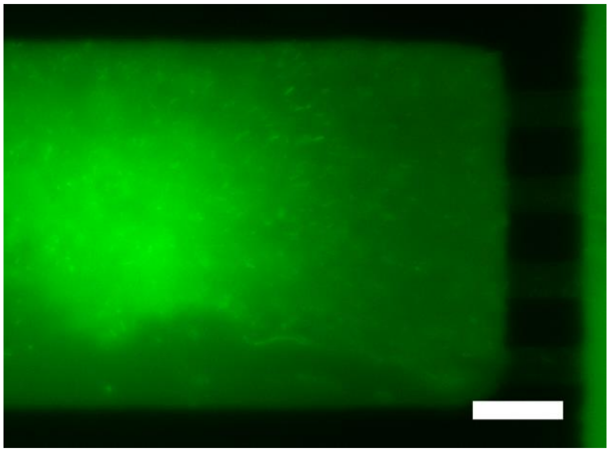

Supplementary Figure 11. Dye penetration indicates penetration barrier is selective. Negatively-charged Alexa Fluor 488 penetrated the chamber in an uninhibited fashion, exhibiting higher levels of intensity towards the interior of the chamber due to the increase in volume as the roof was deformed. Scale bar, 25 µm.

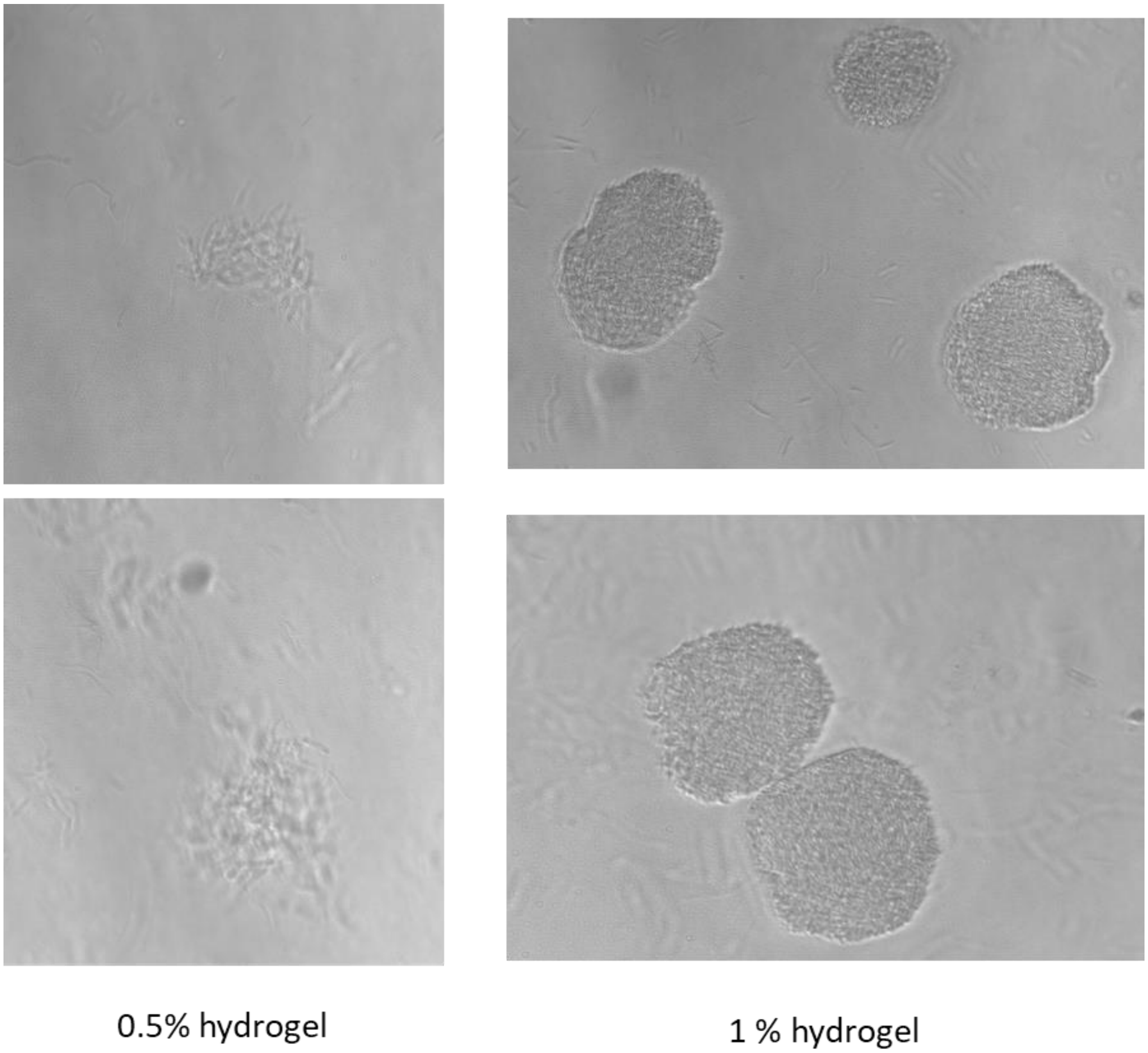

Supplementary Figure 12. Colony growth within hydrogels of various concentrations. Growth of colonies in 0.5% hydrogel resulted in more dispersed and less dense colonies, with fewer cells, whereas 1% hydrogels resulted in dense microcolonies.

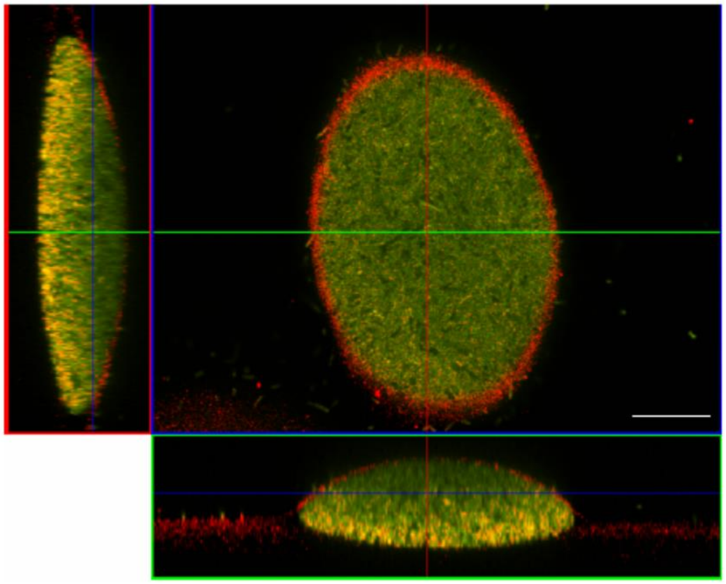

Supplementary Figure 13. 3D reconstruction of colony in hydrogel. Different cross-sections of a colony grown under conditions similar to those shown in Fig. 4b and stained for EPS using rhodamine-labeled concanavalin A. The colony was grown close to the wall of the Petri Dish containing the gel, thus making it asymmetric, and increasing the effective stiffness of the gel on one side of colony. This side displays a more intense EPS staining both at the periphery and inside the colony. Scale bar, 20 μm.

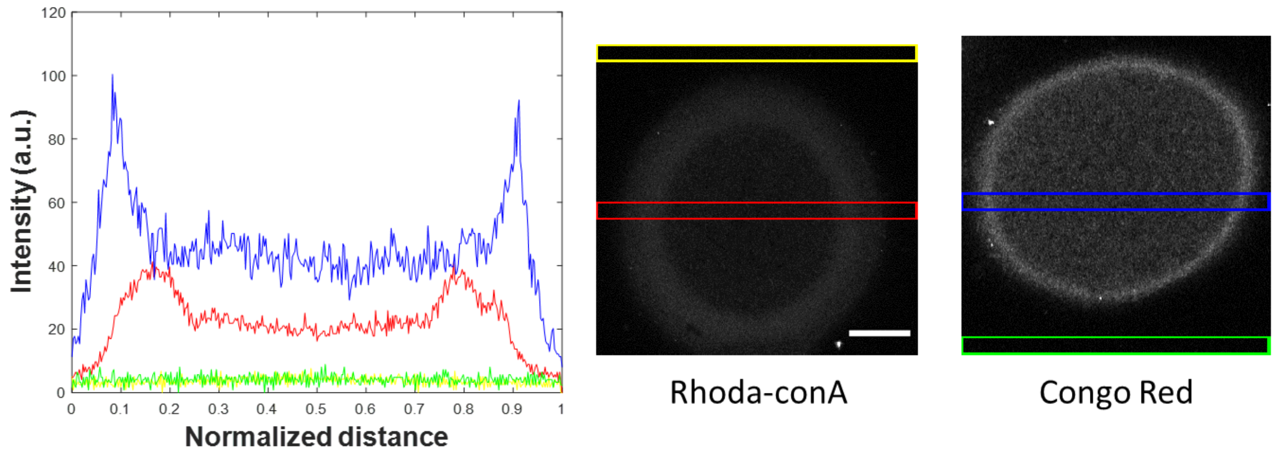

Supplementary Figure 14. Expression of EPS and Curli in hydrogel colonies. Analysis of the micrographs shown in Fig. 4b reveals staining within the interior of microcolonies, demonstrating the expression of EPS and curli inside of the microcolony as well as the ability of the stains to penetrate in. Analysis was performed by averaging the intensities within the color-coded box regions along the y-axis and normalizing the x-axis from 0 to 1. Scale bar, 40 µm.

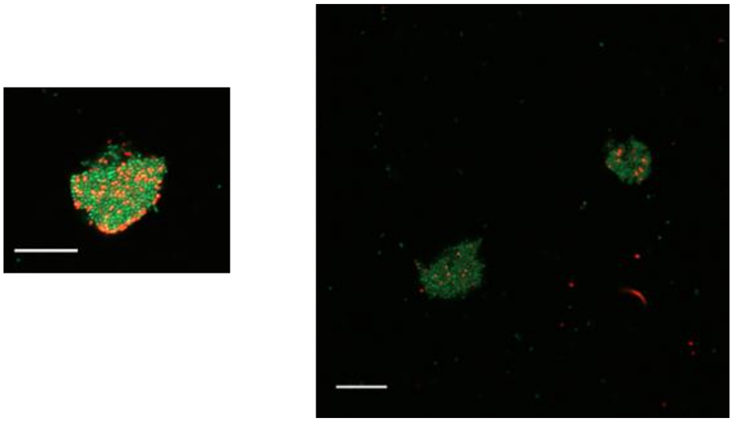

Supplementary Figure 15. Limited antibiotic susceptibility in small colonies. Confocal images of colonies of *E. coli* cells expressing GFP under the control of the *rpoH* promoter forming within the PuraMatrix gel (1%). 6 hrs. of growth followed by a 15 min. long exposure to 10 μg/mL ampicillin and propidium iodide staining showed that many of cells remained intact after the antibiotics treatment. Note the induction of stress response and limited antibiotic susceptibility even at this early stage of colony development. Scale bar, 20 μm.

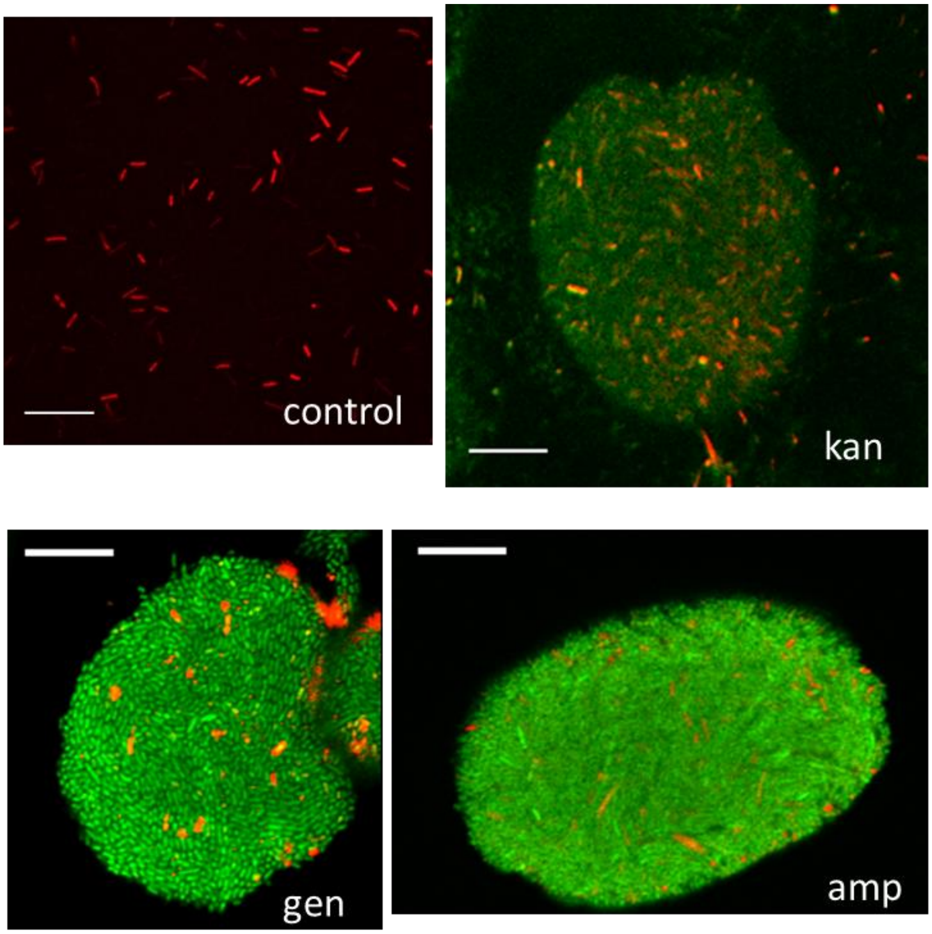

Supplementary Figure 16. Decreased antibiotic susceptibility in hydrogel colonies. Comparison between bulk and hydrogel (6 hr) cultures after exposure to 20 µg/ml of kanamycin (JM105), 20 µg/ml of gentamicin (CFT073), and 10 µg/ml of ampicillin (CFT073), and stained with propidium iodide. Scale bars, 20um.
