# Supplementary material for: Self-induced mechanical stress can trigger biofilm formation in uropathogenic Escherichia coli
Source: Nat Commun. 2018 Oct 5;9:4087. doi: 10.1038/s41467-018-06552-z (PMC6173693; doi:10.1038/s41467-018-06552-z)
Supplement: Supplementary file 2 — Description of Additional Supplementary Files [file 41467_2018_6552_MOESM2_ESM.pdf]

Description of Additional Supplementary Files

File Name: Supplementary Movie 1

Description: 3D rendering of the microfluidic device. The movie shows a 3D render of the chamber array arrangement, with the overlapping pressure channel.

File Name: Supplementary Movie 2

Description: Deformation of chamber membrane from colony growth. The movie shows the deformation of the membrane (black area between blue (Alexa Fluor 647) and green (GFP-expressing E. coli) layers) as a result of colony expansion within confinement. Scale bar, 20 µm.

File Name: Supplementary Movie 3

Description: Colony growth. The movie shows the time-lapse capture of colony growth within chamber. Scale bar, 40 µm.

File Name: Supplementary Movie 4

Description: Z-scan of a full chamber with deformed membrane. The movie shows a confocal z-stack of a fully expanded chamber. Note the "butterfly shape" spatial distribution of GFP as a reporter of rpoH expression. Scale bar, 40 µm.

File Name: Supplementary Movie 5

Description: Simulation of colony in chamber. The movie shows the model simulation of growth and stress response profile within growth chamber with deformable roof.

File Name: Supplementary Movie 6

Description: Simulation of colony in hydrogel. The movie shows the model simulation of growth and stress response profile within hydrogel
